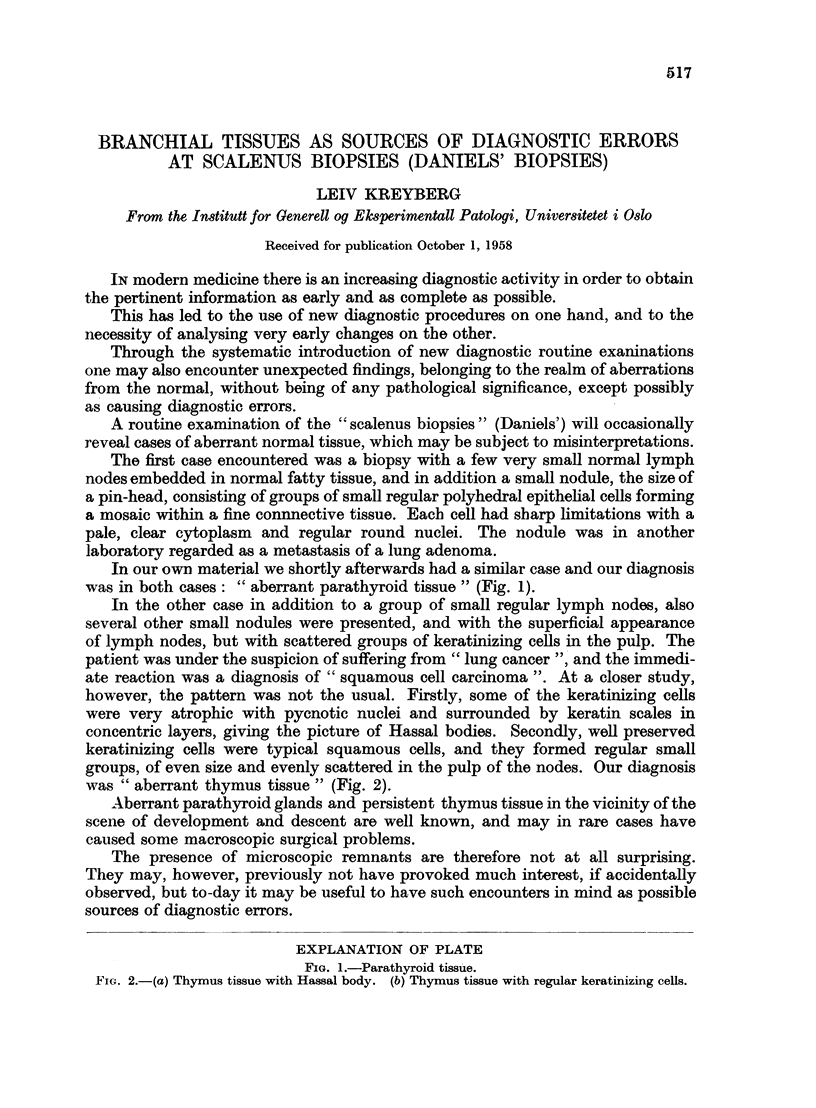# Branchial Tissues as Sources of Diagnostic Errors at Scalenus Biopsies (Daniels' Biopsies)

**DOI:** 10.1038/bjc.1958.59

**Published:** 1958-12

**Authors:** Leiv Kreyberg


### BRANCHIAL TISSUES AS SOURCES OF DIAGNOSTIC ERRORS AT SCALENUS BIOPSIES (DANIELS' BIOPSIES)

LEIV KREYBERG

*From the Institutt for Generell og Eksperimentall Patologi, Universitetet i Oslo*

Received for publication October 1, 1958

IN modern medicine there is an increasing diagnostic activity in order to obtain the pertinent information as early and as complete as possible.

This has led to the use of new diagnostic procedures on one hand, and to the necessity of analysing very early changes on the other.

Through the systematic introduction of new diagnostic routine exaninations one may also encounter unexpected findings, belonging to the realm of aberrations from the normal, without being of any pathological significance, except possibly as causing diagnostic errors.

A routine examination of the "scalenus biopsies" (Daniels') will occasionally reveal cases of aberrant normal tissue, which may be subject to misinterpretations.

The first case encountered was a biopsy with a few very small normal lymph nodes embedded in normal fatty tissue, and in addition a small nodule, the size of a pin-head, consisting of groups of small regular polyhedral epithelial cells forming a mosaic within a fine connnective tissue. Each cell had sharp limitations with a pale, clear cytoplasm and regular round nuclei. The nodule was in another laboratory regarded as a metastasis of a lung adenoma.

In our own material we shortly afterwards had a similar case and our diagnosis was in both cases : " aberrant parathyroid tissue " (Fig. 1).

In the other case in addition to a group of small regular lymph nodes, also several other small nodules were presented, and with the superficial appearance of lymph nodes, but with scattered groups of keratinizing cells in the pulp. The patient was under the suspicion of suffering from " lung cancer ", and the immediate reaction was a diagnosis of " squamous cell carcinoma ". At a closer study, however, the pattern was not the usual. Firstly, some of the keratinizing cells were very atrophic with pycnotic nuclei and surrounded by keratin scales in concentric layers, giving the picture of Hassal bodies. Secondly, well preserved keratinizing cells were typical squamous cells, and they formed regular small groups, of even size and evenly scattered in the pulp of the nodes. Our diagnosis was " aberrant thymus tissue " (Fig. 2).

Aberrant parathyroid glands and persistent thymus tissue in the vicinity of the scene of development and descent are well known, and may in rare cases have caused some macroscopic surgical problems.

The presence of microscopic remnants are therefore not at all surprising. They may, however, previously not have provoked much interest, if accidentally observed, but to-day it may be useful to have such encounters in mind as possible sources of diagnostic errors.

---

EXPLANATION OF PLATE

FIG. 1.—Parathyroid tissue.

FIG. 2.—(*a*) Thymus tissue with Hassal body. (*b*) Thymus tissue with regular keratinizing cells.